# Americas’ opioid/psychostimulant epidemic would benefit from general population early identification of genetic addiction risk especially in children of alcoholics (COAs)

**Published:** 2019-10-31

**Authors:** K Blum, D Baron, M Hauser, S Henriksen, PK Thanos, C Black, D Siwicki, EJ Modestino, BW Downs, S Badgaiyan, TA Simpatico, B Boyett, RD Badgaiyan

**Affiliations:** 1 Western University Health Sciences, Graduate School of Biomedical Sciences, Pomona, CA, USA; 2 Division of Addiction Services, Dominion Digagnostics, LLC., North Kingstown, RI, USA; 3 Division of Nutrigenomics, Geneus Health LLC., San Antonio, TX, USA; 4 Division of Nutrigenomics, Victory International, Inc., Lederach, PA, USA; 5 Department of Psychiatry, University of Vermont, Burlington, VM, USA; 6 Divion of Neuroscience and Addiction Research, Pathway HealthCare, LLC., Birmingham, AL, USA; 7 Behavioral Neuropharmacology and Neuroimaging Laboratory on Addiction, Research Institute on Addictions, University of Buffalo, Buffalo, NY, USA; 8 Claudia Black Young Adult Center, Wickenburg, AZ, USA; 9 Department of Psychology, Curry College, Milton MA, USA; 10 Department of Psychiatry, South Texas Veteran Health Care System, Audie L. Murphy Memorial VA Hospital, San Antonio, TX, Long School of Medicine, University of Texas Medical Center, San Antonio, TX, USA

The United States is in the midst of an opioid overdose epidemic. According to the CDC, from 1999 to 2017, more than 700,000 people have died from a drug overdose (CDC). [Center for Disease Control, Understanding the Epidemic. https://www.cdc.gov/drugoverdose/epidemic/index.html]. In fact, they cite that on average, 130 Americans die every day from an opioid overdose. [Wide-ranging online data for epidemiologic research (WONDER). Atlanta, GA: CDC, National Center for Health Statistics; 2017. Available at http://wonder.cdc.gov]. In 2016, 2.1 million people in the United States suffered from an opioid use disorder (OUD) related to prescription opioids and 262,000 had an OUD related to heroin (CDC) [https://www.cdc.gov/opioids/Medication-Assisted-Treatment-Opioid-Use-Disorder-Study.html]. They also cite that around 68% of the more than 70,200 drug overdose deaths in 2017 involved an opioid. The CDC statistic reported by the National Institute of Drug Abuse estimated that number to be greater than 72,000 [https://www.drugabuse.gov/related-topics/trends-statistics/overdose-death-rates]. Moreover, the CDC reports that in 2017, the number of overdose deaths involving opioids (including prescription opioids and illegal opioids like heroin and illicitly manufactured fentanyl) was 6 times higher than in 1999. Prescription opioid overdose, abuse, and dependence carries high costs for American society, with an estimated total economic burden of $78.5 billion, according to a study published in the October issue of *Medical Care, published by* Wolters Kluwer (CDC). [https://www.sciencedaily.com/releases/2016/09/160914105756.html; https://www.overdoseday.com; https://www.cdc.gov/drugoverdose/index.html]. Goals include increasing awareness about the risk for overdose possibly through genetic testing, reducing stigma associated with drug overdose deaths, providing information about community services, expanding recovery high schools, and preventing and reducing drug-related harm by supporting evidence-based policy and practice [1].

Opioid deaths, particularly those involving illicit opioids, continue to increase. As described in a report of Morbid Mortality Weekly Report (*MMWR)*, illicit opioids were detected in approximately three of four opioid overdose deaths compared with nearly four of 10 for prescription opioids, in the 11 states examined. Enhanced surveillance for opioid overdose deaths facilitates the classification of deaths involving prescription and illicit opioids as well as identifying missed opportunities for prevention and response [2]

It is now in 2019 established that the overall cost of the opioid crisis is north of one trillion dollars. While there are a number of proven strategies available to manage chronic pain effectively without opioids, as well as aberrant drug seeking, it is agreed by all the major agencies that as a unified community, we are being challenged to provide alternative non-addicting and non-pharmacological alternatives to assist in pain and addiction attenuation [3].

While the heroin and opioid epidemic has been front and center in the US, it appears that cocaine is making a comeback. Since 1913 the expansion of Colombia’s illegal coca crop has driven demand on US streets. The 2015 amphetamine users increased globally, reaching 37 million, and new cocaine use expanded to 2 percent of the US population last year. Unlike for opioids and alcohol, there is no FDA medication approval for psychostimulants. However, gene-guided therapy presented herein may be useful in treatment and relapse prevention for abusable drugs [4].

The impact of this global crisis was highlighted by the designation of International Overdose Awareness Day, on August 31, 2018. Extant Addiction research has demonstrated the key to effective treatment is early identification and treatment of drug use. Like cancer, heart disease and other chronic illnesses, the longer they go untreated, the more challenging they are to effectively treat.

It is noteworthy, that a small country such as Iceland with a population of less than one million, actually performs genomic testing at birth for all newborn babies, if parents’ approve. The ability to identify an at-risk population, particularly in childhood would be a major step forward in the attempt to alter the negative impact on individual and population health. Blum’s description of the Reward Deficiency Syndrome (RDS) [5] now featured in SAGE Encyclopedia of Abnormal Psychology (2017) has gained wide acceptance in the scientific community as a critical factor in the etiology of addictive behaviors of all types (drug and non-drug risky behaviors like gaming etc.). In fact, through a Bayesian mathematical approach the Reward Deficiency Syndrome Predictive Value in terms of a child carrying the DRD2 A1 allele is 74.4%.

One example of a special population group that could benefit by early screening in select groups like COAs with Genetic Addiction Risk Score (GARS®) testing is Children of Alcoholics. Claudia Black pioneered the concept of Children of Alcoholics (COAs) in a number of her books (Black, 2018) [6]. Children of alcoholic’s experience suffering with the dysfunction and stress in their home. A child may suffer depression and anxiety, they are also prone to experience difficulty in everyday conditions such as solving problems and making friends. They do not have secure and good role models because their parents cannot find ways to deal or solve their problems. In one study Blum’s group investigated the prevalence of the DRD2A1 allele in COAs. They found that these children showed a significantly greater association with the A1 allele than nonalcoholics but not when compared to alcoholics [7].

This may cause lack of support that can cause a child to do poorly in every activity especially in schools that eventually may affect their characteristics or personality. Understanding this unwanted situation this negative environment-having epigenetic impact in spite of DNA polymorphisms in terms of mRNA expression, loads onto an exaggerated need to secure a “dopamine fix” potentially through abarrant drug and non-drug seeking behavior as observed in RDS [8]. While it is true that more people will become alcoholics based on their genetics [7] it is also true that living with alcoholics also increases the risk. Here are few a few statistics concerning:

- 76 million Americans have alcoholism in their family
- One in four children live with a person that has an alcohol problem
- 33% of any population of alcoholics studied lived with an alcoholic growing up
- 26.8 million Americans are COAs
- 50–60% risk for alcoholism may be due to genetic polymorphisms
- COAs are four times at higher risk to become alcoholics compared to the general population
- First degree of relatives of alcoholics have a 2–4 times greater risk of becoming a n alcoholic in later life
- 90% of innate tolerance to alcohol is inherited.

The risk for future abuse of alcohol, for example, has been highlighted by earlier work by Blum’s group [9] in genetically bred rodent models. Specially, three strains of mice, ICR Swiss, DBA/2J, and C57Bl/6J were compared for initial sensitivity and recovery from intoxication, and acute development of tolerance to ethanol. The alcohol loving C57Bl/6J mice were less sensitive and recovered comparatively quickly at the same dose of ethanol as given to the other two, less loving or hating alcohol strains. In humans, Blum, Noble and associates showed that more Children of Alcoholics (COAs) had a significantly greater association with the DRD2 A1 allele than children of non-alcoholics [7]. In support of this result, Schuckit’s group assessed the risk for alcoholism among sons of alcoholics by measuring tolerance to a single dose of ethanol using a sway machine. Similar to the results of the rodent study by Elston *et al.* [9], these authors also found that the sons of alcoholics had higher innate tolerance to a single dose of ethanol, compared to sons of non-alcoholics. They concluded that a low level of response (LR) to alcohol had been shown to predict a high future risk fo alcoholism. Their robust findings suggested that future research on the relationship between LR and the risk for alcoholism in family history positive (FHPs) can be carried out with a single intoxicating dose of alcohol and without family history, negative (FHN) controls [10]. These data support the concept that family history of SUD or behavioral addictions (like overeating) load onto a high risk for substance misuse in children who may like their parents need treatment. Early intervention and genetic identification may help young adults avoid dangerous substance use and as such have a prophylaxis effect.

The lack of dopamine homeostasis may play a critical role in the development of addictive behaviors, as well as offering effective treatment options. We are proposing a new strategy to help identify at-risk children for the future development of addictions through the use of the validated and USPTO patented Genetic Addiction Risk Score (GARS). Geneus Genomic Testing Center (GGTC), in conjunction with Dominion Diagnostics and Colorado University, in unpublished research (a 5 year sojourn) sought to address genetic risk for alcohol/drug (i.e. opioids) seeking by evaluating the combined effect of reward gene polymorphisms and least 11 polymorphisms and ten genes] contributing to a hypodopaminergic-trait (D1–4, DAT1, Mu opiate receptor, Serotonin transporter, COMT, MAO-A, GABA receptor) as a predictor of severity as measured by the Addiction Severity Index (ASI) Media–Version–V. In unpublished study our test results consisting of 273 mixed gender subjects from seven diverse chemical dependency programs across the United states, show that if a patient carries any combination of 4 genetic risk alleles, it is predictive of drug severity including opioids, or any combination of 7 risk alleles, the test is predictive of alcohol severity (p<0.05, P<0.00 respectively).

As discussed in an earlier paper, genomic testing such as GARS, can improve clinical interactions and decision-making [11]. Knowledge of precise polymorphic associations can help in the attenuation of guilt and denial, corroboration of family gene-o-grams; assistance in risk-severity-based decisions about appropriate therapies, including pain medications and risk for addiction; choice of the appropriate level of care placement (i.e., inpatient, outpatient, intensive outpatient, residential); determination of the length of stay in treatment; determination of genetic severity-based relapse and recovery liability and vulnerability; determination of pharmacogenetic medical monitoring for better clinical outcomes (e.g., the A1 allele of the DRD2 gene reduces the binding to opioid delta receptors in the brain, thus, reducing Naltrexone’s clinical effectiveness); and supporting medical necessity for insurance scrutiny [12–15].

In summary, the ability to identify at-risk children, and follow them prospectively throughout their teenage years, monitoring their drug use/abuse, could have significant public health implications, while expanding our understanding of the core biology of addiction. An epidemic of this size and magnitude will require bold, innovative interventions/selective screenings, educational awareness campaigns that are based on high-quality scientific information and protective monitoring of sensitive DNA data. While appreciating one’s personal choice, rather than just mandating these programs, the authors offer a potential strategy to achieve these important objectives, which merit serious debate especially in COAs.
